# Evaluation of rapid SARS-CoV-2 antigen tests, AFIAS COVID-19 Ag and ichroma COVID-19 Ag, with serial nasopharyngeal specimens from COVID-19 patients

**DOI:** 10.1371/journal.pone.0249972

**Published:** 2021-04-08

**Authors:** Oh Joo Kweon, Yong Kwan Lim, Hye Ryoun Kim, Yoojeong Choi, Min-Chul Kim, Seong-Ho Choi, Jin-Won Chung, Mi-Kyung Lee

**Affiliations:** 1 Department of Laboratory Medicine, Chung-Ang University College of Medicine, Seoul, Republic of Korea; 2 Division of Infectious Diseases, Department of Internal Medicine, Chung-Ang University College of Medicine, Seoul, Republic of Korea; School of Medicine, Tehran University of Medical Sciences, ISLAMIC REPUBLIC OF IRAN

## Abstract

We evaluated the diagnostic accuracy of two newly developed, point-of-care, rapid antigen tests (RATs) for detecting SARS-CoV-2, the AFIAS COVID-19 Ag and the ichroma^TM^ COVID-19 Ag, and investigated antigen kinetics. A total of 200 serially collected nasopharyngeal (NP) specimens from 38 COVID-19 patients and 122 specimens from negative controls were analyzed. Diagnostic sensitivity and specificity were assessed in comparison to molecular test results and subdivided according to targeted genes (*E*, *RdRP*, and ***N***) and days post-symptom onset (PSO). For the kinetics evaluation, cut-off-indices from serial NP specimens were used according to the number of days PSO. Both RATs showed sensitivity of 91.3‒100% for specimens with cycle threshold (Ct) < 25. The specificity of AFIAS was 98.7‒98.9% and that of ichroma^TM^ was 100.0%. The kappa values of AFIAS and ichroma^TM^ for the molecular testing of specimens with Ct < 25 (*RdRP*) were 0.97 and 1.00, respectively. The sensitivity of AFIAS and ichroma^TM^ for all genes was lower for specimens collected at 8‒14 PSO than for those collected before 7-days PSO. The kinetics profiles showed that antigen levels gradually decreased from ≤ 7-days PSO to > 22-days PSO. Both RATs showed excellent specificity and acceptable sensitivity for NP specimens with higher viral loads and for specimens collected within 7-days PSO. Hence, they have the potential to become useful tools for the early detection of SARS-CoV-2. However, because of concerns about false negativity, RATs should be used in conjunction with molecular tests.

## Introduction

The diagnostic reference standard for coronavirus disease 2019 (COVID-19) is the molecular detection of the causative pathogen, severe acute respiratory syndrome coronavirus-2 (SARS-CoV-2) [1–3]. However, it takes 3‒4 h to detect the nucleic acid and requires specialized instruments, expertise, and skilled laboratory personnel who are familiar with molecular techniques [4]. In addition, many countries have encountered a shortage of test kits [5].

Several rapid antigen tests (RATs) were developed for the rapid and inexpensive detection of SARS-CoV-2 and can be used at the point of care (POC). Most RATs are primarily lateral-flow, immunochromatographic assays from nasopharyngeal (NP) swab specimens, which give results in less than 30 min [3, 6]. Although their diagnostic sensitivity for SARS-CoV-2 detection in NP specimens is lower than that of molecular tests [3], the development of a more cost-effective, high-throughput test system with a rapid turnaround time would be important for preventing viral spread.

AFIAS COVID-19 Ag (Boditech Med., Chuncheon-si, Gang-won-do, Republic of Korea) and ichroma^TM^ COVID-19 Ag (Boditech Med.) are POC, immunochromatographic tests designed to detect the SARS-CoV-2 antigen in NP swabs within 20 min. In this study, we evaluated the diagnostic accuracy in terms of sensitivity and specificity of both RATs for SARS-CoV-2 detection using serially collected clinical NP specimens from COVID-19 patients considering real-time RT-PCR (rRT-PCR) as the reference method. In addition, SARS-CoV-2 antigen kinetics was investigated based on the number of days post-symptom onset (PSO).

## Materials and methods

### Clinical specimens

Two hundred NP specimens were collected serially from 38 symptomatic COVID-19 patients (median age 65.5 years, range 24‒91 years, 68.4% male) in Chung-Ang University Hospital, Seoul, Republic of Korea. Among them, 141 and 33 specimens were found to be positive and negative for SARS-CoV-2, respectively, using molecular tests, and the remaining 26 specimens showed inconclusive results. In addition, 122 specimens from non-COVID-19 patients (median age 64.9 years, range 22‒90 years, 67.2% male) were collected. Thus, we analyzed a total of 322 specimens.

### Molecular tests

For molecular determination of the presence of SARS-CoV-2 in NP specimens collected in Virus Transport Medium (VTM, Noble Bio, Hwaseong-si, Gyeonggi-do, Republic of Korea), an automated RNA extraction was carried out using the eMAG^TM^ system (bioMérieux, Inc., Marcy-l’Étoile, France). The extracted RNA was used to perform rRT-PCR with the Allplex^TM^ 2019-CoV Assay (Seegene, Seoul, Republic of Korea) using the real-time PCR instrument CFX96 (Bio-Rad Laboratories, Inc., CA, USA). The target genes were *E*, *RdRP*, and *N* genes. A sample was considered “SARS-CoV-2 positive” when the cycle threshold (Ct) values of all genes in its RNA were < 40. If only one or two of the tested genes had Ct < 40, the test results were interpreted as being “inconclusive” according to the manufacturers’ s instructions [7] and local guidelines [2]. The sample was considered “negative” when the Ct values of all the genes were > 40.

### Antigen tests for SARS-CoV-2

Two immunochromatographic, POC, fluorescence-based RATs—AFIAS COVID-19 Ag and ichroma^TM^ COVID-19 Ag—were evaluated. Residual NP swab specimens in VTM were used, and they were aliquoted and preserved at -70°C until their antigen analysis.

The AFIAS COVID-19 Ag (hereafter referred to as AFIAS) assay is a sandwich fluorescent immunoassay for automatic qualitative/semiquantitative (through signal intensity cut-off index (COI)) determination of SARS-CoV-2 antigens using monoclonal anti-SARS-CoV-2 antibody targeting the nucleocapsid protein within 20 min on human NP swab specimens. For testing, eight drops (about 180 μL) of 1:1 mixtures of NP specimens in VTM with extraction buffer was dispensed into the specimen well on the cartridge containing the test strip. After loading the cartridge into the AFIAS-6 system (Boditech Med Inc.), all procedures from loading the detection buffer into the cartridge to obtaining test results are conducted automatically. Briefly, a fluorescence-labeled antibody conjugate in detection buffer binds to the antigen in a specimen to form antibody-antigen complexes. The complexes migrate onto the nitrocellulose membrane and are captured by anti-SARS-CoV-2 antibody on the test line of the strip. The presence of more antigens in the specimen results in the formation of more antigen-antibody complexes and leads to a fluorescence signal of stronger intensity, which is processed to determine the relative concentrations of SARS-CoV-2 antigens in the specimen. Results were interpreted according to the COI, which was determined by using an equation based on the specimen-to-positive-control signal ratio. COI < 1.0 was interpreted as “negative”, and COI ≥ 1.0 was “positive” for the SARS-CoV-2 antigen [8].

The ichroma^TM^ COVID-19 Ag (hereafter referred to as ichroma^TM^) has an almost identical assay principle as AFIAS, except that it is a manual assay. For testing, six drops of (about 150 μL) 1:1 mixtures of NP swab specimens in extraction buffer were loaded onto the detector tube containing fluorescence-labeled antibody conjugate. 75 μL final mixtures were loaded into the ichroma^TM^ cartridge manually, and after 20 min, the signals from the test line of the strip were read using the signal reader, ichroma^TM^ II (Boditech Med Inc.). Result interpretation criteria were identical to those of AFIAS [9].

### Statistical analysis

To determine the diagnostic accuracy of the two assays of interest, diagnostic sensitivity and specificity were calculated and subdivided by *E*, *RdRP*, and *N* genes and number of days PSO, using 2-by-2 crosstab analysis. Simulated positive predictive values (PPVs) and negative predictive values (NPVs) were calculated according to various prevalence rates of COVID-19. To determine the concordance of diagnostic performance, the Cohen’s kappa (κ) value was used and interpreted according to the criteria proposed by Landis & Koch [10]: bad for values of 0.01–0.20, fair for 0.21–0.40, moderate for 0.41–0.60, strong for 0.61–0.80, and almost perfect for 0.81–1.00. Positivity of rRT-PCR for each gene was inferred for Ct < 40.

For kinetics evaluation, a total of 141 serial NP specimens from 38 confirmed cases of COVID-19 were assessed by following timeframes based on days PSO: < 7 days (32 specimens from 19 patients), 8‒14 days (68 from 33), 15‒21 days (32 from 18), and ≥ 22 days (9 from 6).

For statistical analysis and graphical representation, Microsoft Excel (Microsoft, WA, USA), IBM SPSS® software version 25 (IBM, Armonk, NY), and R version 3.6.1 (http://www.R-project.org) were used.

### Ethics statement

The protocol was approved by the Institutional Review Board (IRB) of Chung-Ang University Hospital (Seoul, Republic of Korea; approval no. 2042-002-412), and written informed consent was obtained from all study subjects.

## Results

### Diagnostic accuracy

Diagnostic sensitivity of two RATs according to targeted gene and Ct values are displayed in Table 1. For *RdRP* and *N* genes, both AFIAS and ichroma^TM^ showed 100.0% sensitivity (95% confidence interval (CI), 90.0‒100.0% for AFIAS/*RdRP* and ichromaTM/*RdRP*, 87.7‒100.0% for AFIAS/*N* and ichroma^TM^/*N*) for specimens with Ct < 25. For *E* gene, the values were 91.3% (79.2‒97.6%) for AFIAS and 95.7% (85.2‒99.5%) for ichroma^TM^. For specimens with Ct values ranging from 25 to 30, the sensitivity of AFIAS and ichroma^TM^ ranged from 34.0% to 64.4%, depending on the target gene. Sensitivity of both antigen assays for specimens with Ct > 30 was even lower than that for the specimens with Ct < 30.

**Table 1:** Diagnostic sensitivity of the AFIAS COVID-19 Ag and the ichroma^TM^ COVID-19 Ag, the rapid point-of-care SARS-CoV-2 antigen assays compared to real-time RT-PCR assay for SARS-CoV-2 detection.

		PCR, *E* gene				PCR, *RdRP* gene				PCR, *N* gene			
	Ct value	<25	25‒30	30‒40	Total	<25	25‒30	30‒40	Total	<25	25‒30	30‒40	Total
AFIAS	Positive (N)	42	16	8	66	35	20	11	66	28	26	12	66
	Negative (N)	4	31	40	75	0	28	62	90	0	19	82	101
	Sensitivity (%)	91.3	34.0	16.7	46.8	100.0	41.7	15.1	42.3	100.0	57.8	12.8	39.5
ichroma^TM^	Positive (N)	44	18	2	64	35	24	5	64	28	29	7	64
	Negative (N)	2	29	46	77	0	24	68	92	0	16	87	103
	Sensitivity (%)	95.7	38.3	4.2	45.4	100.0	50.0	6.9	41.0	100.0	64.4	7.5	38.3

Abbreviations: Ag, antigen; Ct, cycle threshold value; AFIAS, AFIAS COVID-19 Ag (Boditech Med., Chuncheon-si, Gang-won-do, Republic of Korea); ichroma^TM^, ichroma^TM^ COVID-19 Ag (Boditech Med.)

Specificity of AFIAS for *E*, *RdRP*, and *N* genes was 98.9% (179/181, 95% CI 96.1‒99.9%), 98.8% (164/166, 95.7‒99.9%), and 98.7% (153/155, 95.4‒99.8%), respectively, whereas specificity of ichroma^TM^ was 100.0% for all genes (181/181, 98.0‒100.0% for *E*, 166/166, 97.8‒100.0% for *RdRP*, and 155/155, 97.7‒100.0% for *N*). The κ values of AFIAS and ichroma^TM^ compared to the rRT-PCR for the specimens with Ct < 25 (*RdRP*) were 0.97 and 1.00, which was interpreted as “almost perfect” agreement. The κ values among the two antigen assays and rRT-PCR for other genes with these specimens were also higher than 0.92. For all specimens, the κ values were ranged from 0.37 to 0.49 (“fair” ~ “moderate” agreement), according to RATs and target genes.

Table 2 shows the simulated PPVs and NPVs of antigen assays for specimens with Ct < 25 according to disease prevalence. For the setting of 1% of prevalence, PPVs of AFIAS and ichroma^TM^ for *RdRP* gene were 45.7% and 100.0%, respectively. If the prevalence increased to 10%, the values of the assays were 90.3% and 100.0%, respectively. NPVs of both assays for each gene were higher than 99.0% for disease prevalence of 0.5‒10.0%.

**Table 2:** Simulated negative and positive predictive values of the AFIAS COVID-19 Ag and the ichroma^TM^ COVID-19 Ag assays according to the disease prevalence for the SARS-CoV-2 positive nasopharyngeal swab specimens with cycle threshold values less than 25.

	AFIAS						ichroma^TM^					
	PPV (%)			NPV (%)			PPV (%)			NPV (%)		
Prevalence	*E*	*RdRP*	*N*	*E*	*RdRP*	*N*	*E*	*RdRP*	*N*	*E*	*RdRP*	*N*
0.5%	29.4	29.5	27.9	100.0	100.0	100.0	100.0	100.0	100.0	100.0	100.0	100.0
1.0%	45.6	45.7	43.7	99.9	100.0	100.0	100.0	100.0	100.0	100.0	100.0	100.0
2.0%	62.9	63.0	61.1	99.8	100.0	100.0	100.0	100.0	100.0	99.9	100.0	100.0
5.0%	81.4	81.4	80.2	99.5	100.0	100.0	100.0	100.0	100.0	99.8	100.0	100.0
10.0%	90.2	90.3	89.5	99.0	100.0	100.0	100.0	100.0	100.0	99.5	100.0	100.0

Abbreviations: Ag, antigen; AFIAS, AFIAS COVID-19 Ag (Boditech Med., Chuncheon-si, Gang-won-do, Republic of Korea); ichroma^TM^, ichroma^TM^ COVID-19 Ag (Boditech Med.); PPV, positive predictive value; NPV, negative predictive value

**Fig 1:**
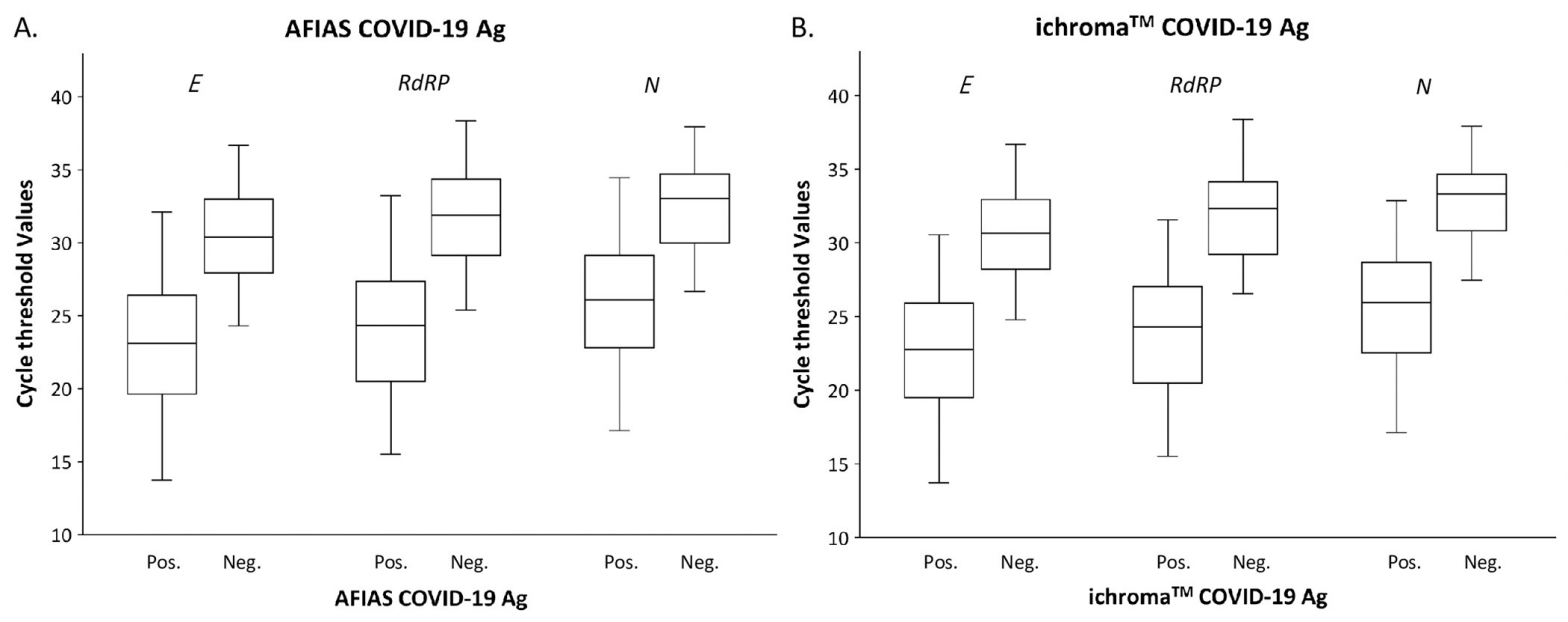
Cycle threshold value distribution of *E*, *RdRP*, and *N* genes according to the positivity of (A) the AFIAS COVID-19 Ag and (B) the ichroma^TM^ COVID-19 Ag assays. Abbreviations: Ag, antigen; PSO, Pos.; positive; Neg., negative.

The distribution of Ct values of each gene according to the antigen test positivity is displayed in Fig 1. Antigen-positive specimens had significantly lower Ct values than antigen-negative specimens. Median Ct values for *E*, *RdRP*, and *N* genes of PCR-positive/AFIAS-positive specimens were 23.10 (min‒max, 13.72‒32.11), 24.34 (15.52‒33.22), and 26.08 (17.13‒34.45), respectively, and the values for PCR-positive/AFIAS-negative specimens were 30.39 (24.30‒36.69), 31.88 (25.40‒38.37), and 33.02 (26.65‒37.93), respectively. The values for PCR-positive/ichroma^TM^-positive specimens were 22.77 (13.72‒30.54), 24.30 (15.52‒31.57), and 26.00 (17.13‒32.87), respectively, and those for PCR-positive/ichroma^TM^-negative specimens were 30.66 (24.79‒36.69), 32.32 (26.56‒38.37), and 33.33 (27.46‒37.93), respectively.

Table 3 shows the diagnostic sensitivity of the antigen tests according to the number of days PSO. For the specimens collected at ≤ 7-days PSO, sensitivity of AFIAS and ichroma^TM^ was 84.4% (95% CI, 67.2‒94.7%) and 81.3% (63.6‒92.8%) for all genes, respectively. For the specimens collected at 8‒14-days PSO, the sensitivity values for AFIAS ranged from 42.1% to 47.1%, and the values for ichroma^TM^ ranged from 47.4% to 52.9%. Much lower sensitivity (<17.1%) was observed for specimens collected after 14-days PSO than for specimens collected before this timeframe.

**Table 3:** Diagnostic sensitivity of the AFIAS COVID-19 Ag and the ichroma^TM^ COVID-19 Ag assays according to the days post-symptom onset.

		≤ 7 days PSO (Ct < 40 / < 25)			8–14 days PSO (Ct < 40 / < 25)			>14 days PSO (Ct < 40 / < 25)		
		*E*	*RdRP*	*N*	*E*	*RdRP*	*N*	*E*	*RdRP*	*N*
AFIAS	Positive, N	27 / 21	27 / 19	27 /17	32 / 21	32 / 16	32 / 11	7 / 0	7 / -^a^	7 / -
	Negative, N	5 / 0	5 / 0	5 / 0	36 / 2	42 / 0	44 / 0	34 / 2	43 / -	52 / -
	Sensitivity, %	84.4 / 100.0	84.4 / 100.0	84.4 / 100.0	47.1 / 91.3%	43.2 / 100.0	42.1 / 100.0	17.1 / 0.0	14.0 / -	11.9 / -
ichroma^TM^	Positive, N	26 / 21	26 / 19	26 / 17	36 / 23	36 / 16	35 / 11	2 / 0	2 / -	2 / -
	Negative, N	6 / 0	6 / 0	6 / 0	36 / 0	38 / 0	40 / 0	39 / 2	48 / -	57 / -
	Sensitivity, %	81.3 / 100.0	81.3 / 100.0	81.3 / 100.0	52.9 / 100.0	48.7 / 100.0	47.4 / 100.0	4.9 / 0.0	4.0 / -	3.4 / -

Abbreviations: Ag, antigen; PSO, post-symptom onset; Ct, cycle threshold value; AFIAS, AFIAS COVID-19 Ag (Boditech Med., Chuncheon-si, Gang-won-do, Republic of Korea); ichroma^TM^, ichroma^TM^ COVID-19 Ag (Boditech Med.)

a. There were no specimens with *RdRP* and *N* Ct < 25, which were collected after 14-days PSO.

Among the specimens collected before 14-days PSO, the specimens with Ct < 25 showed much higher sensitivity, 91.3‒100.0%, than those with higher Ct values. After 14-days PSO, only two specimens had Ct < 25 for *E* gene only (24.79 and 24.93, respectively), and they were all negative from both antigen tests.

### Antigen kinetics

**Fig 2:**
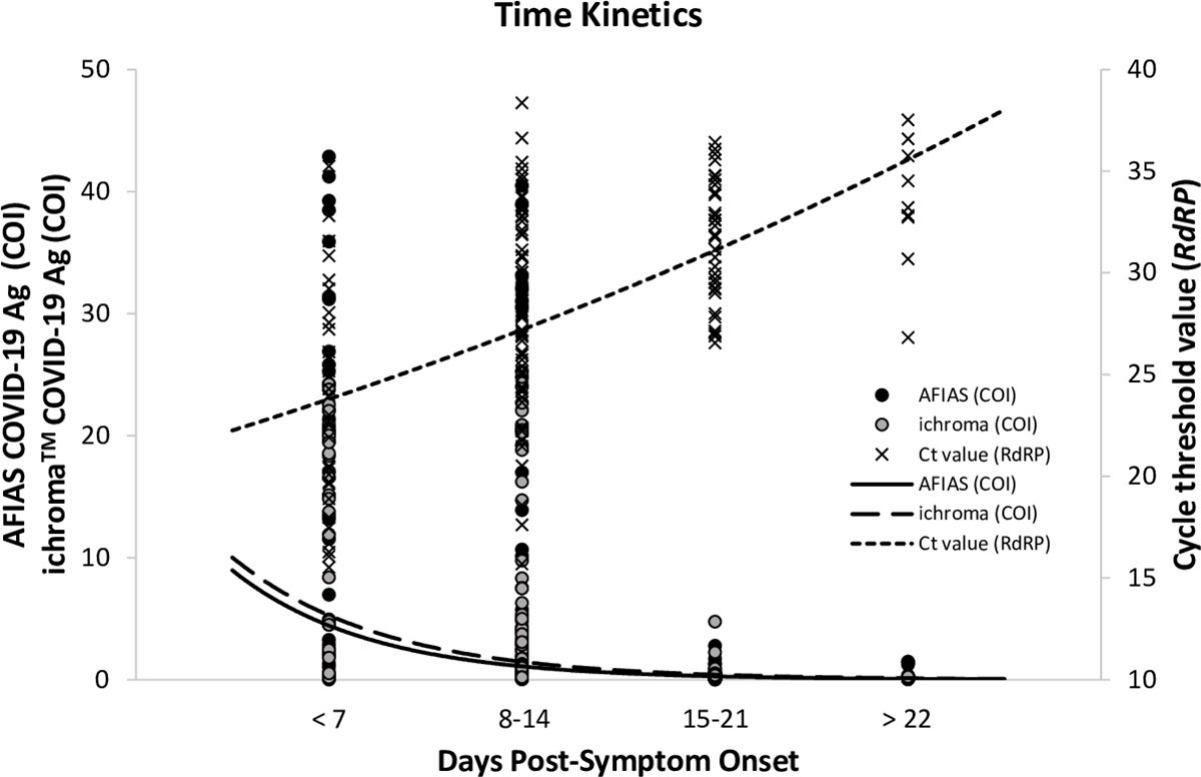
Antigen kinetics determined for specimens from 38 COVID-19 patients at different post-symptom-onset periods assessed by two antigen assays according to cut-off index values, the AFIAS COVID-19 Ag and the ichroma^TM^ COVID-19 Ag. The smoothing splines of both assays showed gradual decrement from the days 1‒7 to days >22, while it was shown that their *RdRP* gene cyclic threshold values gradually increased or their viral loads decreased. Abbreviations: Ag, antigen; COI, cut-off index; AFIAS, AFIAS COVID-19 Ag (Boditech Med., Chuncheon-si, Gang-won-do, Republic of Korea); ichroma, ichroma^TM^ COVID-19 Ag (Boditech Med.); Ct, cycle threshold.

Fig 2 shows the antigen kinetics of samples collected from 38 COVID-19 patients at different PSO periods assessed by two antigen assays according to the COI values. The smoothing splines of both assays showed gradual decrease from days < 7 to days ≥ 22, as it was shown that their *RdRP* gene Ct values gradually increased or that their viral loads decreased. For AFIAS, the median COI values of the specimens collected at < 7 days PSO, 8‒14 days, 15‒21 days, and ≥ 22 days PSO were 13.3 (min‒max, 0.02‒42.89), 0.76 (0.04‒52.91), 0.21 (0.01‒2.77), and 0.07 (0.03‒1.48), respectively. For ichroma^TM^, the median COI values of those specimens were 14.97 (0.16‒24.23), 1.12 (0.12‒25.27), 0.26 (0.06‒4.75), and 0.21 (0.07‒0.33), respectively.

## Discussion

We evaluated the diagnostic accuracy of the rapid SARS-CoV-2 antigen tests, the AFIAS and ichroma^TM^, for the detection of SARS-CoV-2 in NP specimens compared to that of rRT-PCR as the gold standard. In addition, antigen kinetics were examined according to the number of days PSO.

In the pandemic context of COVID-19, diagnostic testing for SARS-CoV-2 is crucial to limit the spread of the virus as well as to effectively manage infected patients. Studies have shown that POC-based RATs have higher limits of detection and therefore, lower sensitivity than molecular tests [11–14]. This leads to concerns about false negative results for specimens with low viral load, despite which RATs can be easy, rapid, and cost-efficient approaches for SARS-CoV-2 detection.

In this study, both RATs showed excellent specificity (98.7‒100.0%) and fairly good sensitivity for specimens with high viral loads (Ct values < 25, 91.3‒100.0% according to the target gene) and for specimens collected before 7-days PSO (81.3‒84.4%). Thus, RATs for NP swabs can be promising tools for the rapid detection of SARS-CoV-2 for patients in the initial phase of symptomatic infection and/or with high viral loads. However, sensitivity for specimens with lower viral loads and for specimens collected after 7-days PSO gradually decreased, and antigen kinetics showed a more acute decrease in COI for both RATs with an increase in Ct values, which was consistent with the results of previous studies [11, 14, 15]. Moreover, several studies reported that NP specimens with Ct > 25 do permit recovery of virus from viral culture, and this is possible even for Ct > 35 [16]. Consequently, negative results from RATs cannot exclude SARS-CoV-2 virus infection confidently and must be verified by further rRT-PCR testing.

As PPV and NPV are significantly affected by disease prevalence, these values should be calculated for various levels of prevalence. Although the PPV of the AFIAS in a 0.5% prevalence setting was significantly low, the PPV in a 10.0% setting was ~90%, which was acceptable. Moreover, the fact that the prevalence of other respiratory viruses is currently even lower than that observed previously, because of various precautions taken to block their spread [17], increases the PPV further in the symptomatic patient population because of which the prevalence of COVID-19 may be expected to rise.

This study has some limitations. First, we employed a modified specimen-processing method to perform the RATs. Although the manufacturers of the RATs recommend the direct use of NP specimens, that approach was not adopted because of certain practical issues. Given that the dilution factor of our processing method (mixture with VTM and buffer) was calculated as about 1:13.4 compared to direct specimens, the diagnostic sensitivity for the specimens with 25 ≤ Ct < 30 would be higher if they were analyzed without dilution. In addition, we used frozen VTM specimens instead of fresh ones for the same reason. The internal validation with borderline specimens did not reveal any discrepancy or significant changes in the test results between fresh and frozen specimens. Moreover, the manufacturers argued that there were no effects of test performance from one cycle of freezing/thawing procedure. However, the sample number for our internal validation was relatively small and vigorous studies focusing on this issue using these RATs were not conducted. On the basis of the fact that even one cycle of freezing/thawing could decrease the viral loads in VTM [18], the possibility that the diagnostic accuracy was adversely affected by using frozen specimens cannot be ruled out. Another limitation was that we did not assess viral loads directly (such as copies/mL in units) and instead, indirectly expressed the viral loads as Ct values. However, as clinical communication about viral loads mainly involves Ct values, it may be more practical to express these loads in Ct values. Another limitation was that we did not perform viral culture, plaque assay, or tissue culture infective dose_50_ assay due to lack of facilities. Especially, viral culture could give us comprehensive information about the specimens that showed discrepant results between RATs and molecular tests. Furthermore, we did not assess the SARS-CoV-2 variant. Both RATs target the nucleocapsid protein of SARS-CoV-2, and variants are known to have key mutations mainly in the spike protein coding region of their RNA sequences [19, 20]. Thus, the new variants are not likely to affect the RATs performances, however, vigorous evaluations with the variants are still needed.

In conclusion, both RATs showed excellent specificity and acceptable sensitivity for the NP specimens with higher viral loads and/or the specimens collected within 7-days PSO. The assays are easy to use, cost-efficient, and most importantly, provide results in a rapid manner. Therefore, they have the potential to become useful tools for the early detection of SARS-CoV-2. However, because of the concerns about false negative results, RATs should be used in conjunction with molecular tests, especially for excluding SARS-CoV-2 infection.
